# Portal vein thrombosis as extraintestinal complications of Crohn’s disease: a case report and review of literature

**DOI:** 10.1186/s13256-024-04560-w

**Published:** 2024-05-14

**Authors:** Marouf Alhalabi, Duaa Nasri, Widad Aji

**Affiliations:** Gastroenterology Department, Damascus Hospital, Almujtahed Street, Damascus, Syria

**Keywords:** Crohn’s disease, Ulcerative colitis, Portal vein thrombosis, Inflammatory bowel disease, Extraintestinal manifestations, Case report

## Abstract

**Introduction:**

Thrombotic events are more than twice as common in inflammatory bowel disease patients as in the general population. We report an interesting and rare case of portal vein thrombosis as a venous thromboembolic event in the context of extraintestinal manifestations of Crohn’s disease. We also conducted a literature review on portal vein thrombosis associated with inflammatory bowel disease, with the following concepts: inflammatory bowel diseases, ulcerative colitis, Crohn’s disease, portal vein, and thrombosis.

**Case presentation:**

A 24-year-old Syrian female with active chronic Crohn’s disease was diagnosed 11 years ago and classified as A1L3B1P according to the Montreal classification. She had no prior surgical history. Her previous medications included azathioprine and prednisolone. Her Crohn’s disease activity index was 390 points. Gastroduodenoscopy revealed grade I esophageal varices, a complication of portal hypertension. Meanwhile, a colonoscopy revealed several deep ulcers in the sigmoid, rectum, and descending colon. An investigation of portal vein hypertension revealed portal vein thrombosis. We used corticosteroids to induce remission, followed by tapering; additionally she received ustekinumab to induce and maintain remission. She began on low-molecular-weight heparin for 1 week, warfarin for 3 months, and then apixaban, a novel oral anticoagulant, after excluding antiphospholipid syndrome. Primary prophylaxis for esophageal varices was not required. After 1 year, she achieved clinical, biochemical, and endoscopic remission. Despite 1 year of treatment, a computed tomography scan revealed no improvement in portal vein recanalization.

**Conclusion:**

Portal vein thrombosis is a rare and poorly defined complication of inflammatory bowel disease. It is usually exacerbated by inflammatory bowel disease. The symptoms are nonspecific and may mimic a flare-up of inflammatory bowel disease, making the diagnosis difficult. Portal vein Doppler ultrasound for hospital-admitted inflammatory bowel disease patients may contribute to the diagnosis and management of this complication.

## Introduction

Extraintestinal manifestations can affect almost any organ system and have a negative impact on the patient’s functional status and quality of life. Extraintestinal manifestations are most commonly observed in the joints, skin, hepatobiliary tract, eyes, heart, pancreas, and vascular system. Portal vein thrombosis (PVT) is an obscure and poorly defined complication of many diseases, including cirrhosis, intraabdominal infection, intraabdominal surgery, pancreatitis, primary hematologic disorders, and inflammatory bowel disease (IBD) [1]. The prevalence of PVT in patients with IBD ranges from 0.17% to 1.7% [1], and may be associated with inherited or acquired hypercoagulability risk factors and has a benign outcome [1]. It can be difficult to diagnose PVT in patients with IBD because its extremely generic symptoms, such as abdominal discomfort, can frequently originate from any of its triggering events. Therefore, it should come as no surprise that the diagnosis is frequently made by accident when imaging is performed to check for one of these triggering processes, also the laboratory results are nonspecific [1]. We report an interesting and uncommon case of PVT associated with Crohn’s disease that was discovered when investigating the cause of esophageal varices related to portal vein hypertension. We also conducted a literature review on portal vein thrombosis associated with inflammatory bowel disease using the following concepts: inflammatory bowel disease, ulcerative colitis, Crohn’s disease, portal vein, and thrombosis.

## Case report

**Fig. 1:**
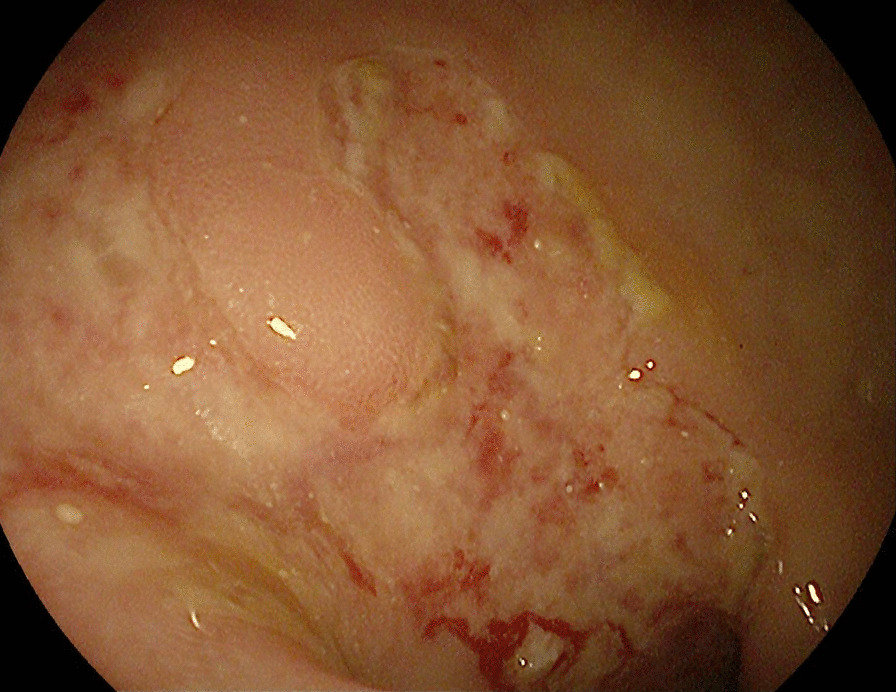
Colonoscopy revealed several ulcerations in the sigmoid, rectum, and descending colon

**Fig. 2:**
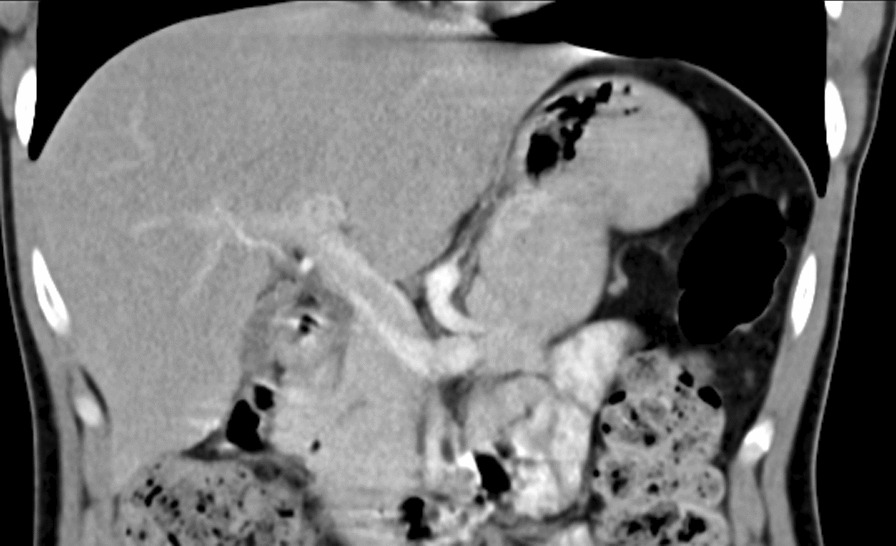
The contrast-enhanced computed tomography scan of the abdomen, which shows portal vein thrombosis

We evaluated a 24-year-old Syrian female with active chronic Crohn’s disease, diagnosed 11 years ago. She was classified as A1L3B1P according to the Montreal classification [2]. She had no prior surgical history; her past medications included azathioprine 2.5 mg/kg/day since diagnosis until now and prednisolone 1 mg/kg up to 40 mg during flares, then tapering [3]. Furthermore, she did not use oral contraceptive pills. Her weight was 50 kg, her height was 161 cm, and she had a body mass index of 19.29 kg/m^2^. She complained of watery, bloody diarrhea up to eight times a day, accompanied by abdominal pain in the prior month. Her Crohn’s disease activity index (CDAI) was 390 points. Initial blood tests confirmed leukocytosis, anemia, elevated fecal calprotectin (FC), and C-reactive protein (CRP) levels. Stool cultures, *Clostridium difficile* toxin, *Escherichia coli*, and *Cryptosporidium*, as well as microscopy for ova and parasites, all returned negative. The hypercoagulability work-up revealed negative results for anti-Beta-2 Glycoprotein-1 IgM antibodies, antinuclear antibodies (ANA), fibrinogen, protein S (activity), antithrombin III, and homocysteine, whereas lupus anticoagulant (LA1, LA2) was positive. Factor II mutation and factor V Leiden mutation were normal, whereas the methylenetetrahydrofolate reductase mutation was a homozygous mutant gene. The portal system and suprahepatic vein ultrasound revealed a thrombus that covered nearly half of the lumen of the portal vein and splenomegaly. Gogastroduodenoscopy showed grade I esophageal varices (less than 5 mm, without bleeding risk signs), which indicate portal vein hypertension owing to splenomegaly and esophageal varices. In light of the patient’s recent onset of abdominal pain and the absence of portosystemic collaterals on Doppler ultrasound, a recent PVT is a strong possibility [4]. The colonoscopy revealed several deep ulcers in the sigmoid, rectum, and descending colon Fig. 1. The biopsies were negative for *Clostridium difficile*, and immunohistochemical staining was negative for cytomegalovirus (CMV) [3, 5]. The median liver stiffness measured by FibroScan was 2.4 kPa, which suggests the absence of fibrosis. Protein electrophoresis was normal. The abdomen and pelvis contrast-enhanced computed tomography (CT) scan confirmed the PVT and displayed thickening in the descending colon (Fig. 2). Antiphospholipid syndrome was initially diagnosed on the basis of an antiphospholipid profile, a history of PVT (thrombotic event), and an association with Crohn’s disease [6]. She initially received corticosteroids to achieve disease remission, followed by ustekinumab to induce and maintain therapy (390 mg intravenous induction followed by 90 mg subcutaneous every 8 weeks) owing to moderate-to-severe Crohn’s disease unresponsive to azathioprine [7, 8]. She began on low-molecular-weight heparin (LMWH) for 1 week, and warfarin for 3 months with an international normalized ratio (INR) target of 2–3. The lupus anticoagulant (LA1, LA2) was retested after 12 weeks and returned to negative [6], so we switched to apixaban, a novel oral anticoagulant (NOAC) [4]. The 1-year reevaluation indicated clinical, biochemical, and endoscopic remission with CDAI of 150 points, normal lab test, and normal endoscopy. The patient’s tests are presented in Table 1. Despite 1 year of treatment, a CT scan revealed no improvement in portal vein recanalization. We continued 90 mg of subcutaneous (SC) ustekinumab every 8 weeks, while we stopped apixaban [3, 4].

**Table 1:** Patient’s tests

Test	On admission	1 year later	Normal limits	Unit
WBC	13,200	6300	4500–10500	/mm^3^
Hemoglobin	9.9	11.2	12–16	g/dl
Platelets	73	119	150–450	_X_ 1000 mm^3^
Urea	28	19	15–54	mg/dl
Creatinine	0.11	0.13	0.5–1.3	mg/dl
ANA	Negative		< 1/40	titer
ASMA	Negative		< 1/20	titer
AMA-M2	Negative			titer
CRP	36	5	Up to 6	mg/l
Fecal calprotectin	520	49	< 150	µg/g
ALT	11	13	5–45	U/L
AST	35	20	8–40	U/L
Total bilirubin	0.8	0.9	0.5–1.2	mg/dl
Direct bilirubin	0.2	0.2	0–0.3	mg/dl
Total protein	7.2	7.5	6.2–8	g/dl
Albumin	4.2	4.6	3.8–5.1	g/dl
INR	1.2	1		
Glucose	89	86	74–106	mg/dl
Uric acid	3.2	2.9	2.5–6.5	mg/dl
HBsAg	Negative			
Anti-HCV	Negative			
Anti beta-2 glycoprotein-1 IgM antibodies	< 3		< 12	U/mL
Antinuclear antibodies (ANA) Hep-2 cells and primate liver by IFA	Negative		Up to 1/40	
Fibrinogen	304.1	280	180–380	mg/dl
Lupus anticoagulant				
LA 1	84.8	Negative	31–44	Sec
LA2	70.7	Negative	30–38	Sec
Protein S (activity)	147	115	60–150	%
Antithrombin III (activity)	95		60–150	%
Homocysteine (total)	9.86		4.6–12.5	umol/L
Thyroid-stimulating hormone	1.76		0.27–4.2	uIU/ml
Factor II mutation	Normal			
Factor V Leiden mutation	Normal			
Methylenetetrahydrofolate reductase mutation	Homozygous mutant gene			

*WBC* white blood cells, *ANA* antinuclear antibody, *CRP* C-reactive protein, *ALT* alanine aminotransferase, *AST* aspartate aminotransferase, *INR* international normalized ratio, *HbsAg* hepatitis B surface antigen, *Anti-HCV* hepatitis C antibody, *HbcAb* hepatitis B core antibody

## Review of literature

### Methods

To facilitate this literature review, we used a combination of keywords and database subject headings to search the MEDLINE (through PubMed) database on 1 July 2023 for the following concepts: Crohn’s disease, ulcerative colitis, IBD, portal vein, PVT, and thrombosis. We also manually searched the reference lists of the included papers. We returned the research on 7 April 2024, and no new findings were obtained.

### Eligibility criteria

We searched for any case reports, case series, observational, or interventional studies that addressed portal vein thrombosis associated with inflammatory bowel disease. Table 2 summarizes the basic features and treatment outcomes of the reported cases.

**Table 2:** Summary of the basic features of the reported cases

Ref.	Study type	Age	Sex	IBD-status	Hypercoagulability study	Hypercoagulability study result	Treatment	Outcome
Nguyen 2021 [18]	Case report	38	M	CD-flare	Antithrombin III, protein C, protein S, factor V Leiden, prothrombin gene mutation, factor VII, factor VIII, antiphospholipid antibodies, and JAK2 V617F mutation	Negative	Heparin apixaban	Unknown cause of death
Naymagon 2021 [1]	Retrospective cohort	Median:42 (29–55)	M:40/63(63.5%)	UC:37/63(58.7%) Flare:21/63(33%)	Factor V Leiden mutation(2/27), prothrombin gene mutation (1/27), APLS testing (1/27), protein C deficiency (0/24), protein S deficiency (0/24), antithrombin III deficiency (0/20), JAK2V617F mutation (0/16), paroxysmal nocturnal hemoglobinuria (0/6)(	Reported in previous column as positive/number of testing	Direct oral anticoagulant (DOAC) *n* = 23, apixaban (*n* = 4), dabigatran (*n* = 4), rivaroxaban (*n* = 15), warfarin (*n* = 22), and enoxaparin, (*n* = 13)	2 patients died 5/63 patients had small bowel ischemia 45/63 patients achieved CRR of PVT
Ma 2015 [19]	Case report	27	M	CD-Active	Not reported	Not reported	LMWH warfarin	Recanalization of portal venous thrombus
Mian 2015 [20]	Case report	41	F	UC-flare in past 2 weeks	Not reported	Not reported	Heparin Rivaroxaban	Hospital discharged
Landman 2013	Retrospective cohort	Median: 41 (19–69)	M:29/50(58%)	CD: 36/50(72%) Active:32/50 (65%)	Protein C deficiency, protein S deficiency, antiphospholipid syndrome, antithrombin deficiency, factor II gene mutation, hyperhomocysteinemia, factor V Leiden mutation, Jak2 mutation	No clear result were reported	44 (88%) patients were given, mostly vitamin K antagonists	Recanalization rate *n* (%) 27/44 (61)
Maconi* 2012 [21]	Case series	Median: 49 (35−59)	M:5/8 (62.5%)	CD:6/8 (75%) Active:4/8(50%)	Antithrombin, protein C and S activity, plasma homocysteine level, and antiphospholipid antibody testing, including anticardiolipin antibodies and lupus anticoagulant testing. Testing for factor V Leiden, prothrombin, and methyltetrahydrofolate reductase (MTHFR)	One risk factor for hypercoagulability was identified in five patients	Four patients required anticoagulant (unknown)	Varices: two patients Recanalization in all patients
Georgescu 2010 [22]	Case report	42	M	CD Inactive	Protein C,protein S antithrombin activity, homocystein, antiphospholipid antibodies, lupus anticoagulant, anti-beta2 glycoprotein 1, antiphosphatydilserine, antiprothrombin antibodies, anticardiolipin antibody	Positive anticardiolipin antibody,and factor V Leiden	Not reported	Not reported
McCabe [23]	Case report	35	F	CD-Active	Prothrombin, factor V Leiden, anticardiolipin antibody, lupus anticoagulant, antiproteinase 3 antibody, myeloperoxidase antibody, double-stranded DNA antibody, smooth muscle antibody, ANCA, MTHFR mutation, protein C, protein S	Negative	Heparin Warfarin	CD remission
Di Fabio 2009 [24]	Case report	62	F	UC-flare	Not reported		Tissue plasminogen activator (TPA) Heparin	Total proctocolectomy, ileostomy, splenectomy distal pancreatectomy
Racine 2008 [25]	Case report	59	M	CD-InActive	Hyperhomocysteinemia, an antiphospholipid antibody syndrome, factor V Leiden mutation, factor II mutation, JACK 2 mutation, protein S, protein C, and antithrombin III deficiency	Hyperhomocysteinemia, antiphospholipid antibody syndrome	Not reported	Not reported
Babyatsky 2007 [26]	Case report	30	M	UC-Flare	Standards tests	Anticardiolipin antibody IgM	Warfarin	Thrombus resolving
Palkovits 2007 [27]	Case report	35	F	UC-Flare	Factor V Leiden, prothrombin G20210A mutation, activity of coagulation factors and natural coagulation inhibitors, hyperhomocysteinemia, and lupus anticoagulans	Normal	LMWH then stopped owing to gastric varices bleeding risk	Gastric varices bleeding risk
Brueck 2006 [28]	Case report	23	F	UC-flare	Not reported	N/A	Urokinase, heparin, oral anticoagulation	Recovered completely
Shaked 2005 [29]	Case report	23	F	CD-Flare	Anti-thrombin III, protein C, protein S, and factor V Leiden deficiency, and antiphospholipids syndrome	Negative	Heparin Warfarin	Recanalization
Guglielmi 2005 [30]	Case report	40	F	UC-flare	Antiphospholipid antibodies	Negative	Alteplase- (rt-PA)	Rectal bleeding and cerebral hemorrhage. complete resolution of the superior mesenteric and main portal vein thrombosis
Tomita 2005 [31]	Case report	37	M	UC-flare	Protein S, protein C	Negative	Warfarin	Recanalization
Valera 2004 [32]	Case report	29	M	UC-active	Protein C, antithrombin III, factor V, homocysteine	Negative	Heparin Acenocoumarol	Discharged
Ec 2004 [33]	Case report	56	M	UC-inactive	Study for hypercoagulable state	Negative	None	Complete atrophy of the left hepatic lobe secondary to the persistence of the left portal vein thrombosis
Mijnhout 2004 [34]	Case report	42	M	UC-inactive	Not performed	N/A	Heparin Oral anticoagulation	Recanalization
Kluge 2003 [35]	Case report	30	M	CD-inactive	Not performed	N/A	Enoxaparin, surgery	Recanalization
Junge 2001 [36]	Case report	17	F	UC-flare	Protein C, anticardiolipin antibodies, beta 2-glycoprotein antibody, lupus anticoagulant, factor V Leiden, factor XIII, protein C deficiency, and antiphospholipid antibodies	Protein C deficiency and antiphospholipid antibodies	Urokinase, anticoagulation	Orthotopic liver transplantation
Hagimoto 2001 [37]	Case report	38	F	UC-flare	factor VII, antithrombin lI level protein C, protein S	Negative	None	Recanalization
Schäfer 2000 [38]	Case report	29	M	CD-inactive	Not reported	N/A	Urokinase and heparin	Dissolution of main stem thrombus whereas right branch of portal vein remained occluded
Farkas 2000 [39]	Image	32	M	UC, no other information	Not reported	Not reported	Not reported	Not reported
Baddley 1999 [40]	Case report	41	M	CD-inactive	Not reported	Not reported	Not reported	Right colon and terminal ileum resection discharged
Yada 1998 [41]	Case report	35	M	CD-flare	Not reported	Not reported	Not reported	Esophageal varices
Zoepf 1997 [42]	Case report	27	M	CD-no information	No information	No information	No information	No information
Tung 1996 [43]	Case report	18	F	CD-active	Protein S, protein C, fibrinogen, antithrombin III	Negative	Warfarin, heparin	Recanalization
Tsujikawa 1996 [44]	Case report	44	M	CD-inactive	Protein S level, protein C level, and antithrombin III	Negative	Tissue plasminogen activator, urokinase,heparin,	Improvement of portal venous circulation CD remission
Diehl 1996 [45]	Case report	No information	M	CD-inactive	No information	No information	No information	No information
Miyazaki 1995 [46]	Case report	No information	No information	UC-active	No information	No information	No information	No information
Mathieu 1994 [47]	Case report	29	F	CD	Antithrombin III, protein C, protein S homocystinuria, anticardiolipid antibodies, anti-b&a2 GP1, polynuclear anticytoplasm	Polynuclear anticytoplasm, protein S	Fraxiparine	No complication of portal hypertension
Crowe 1992 [48]	Case report	42	M	CD-inactive	Not done	N/A	Warfarin	No complication
Brinberg 1991 [49]	Case report	40	M	CD-flare	Antithrombin III, protein S, and protein C deficiency	Negative	Subcutaneous heparin	No complication
Reh 1980 [50]	Case report	25	M	UC	Not reported	Not reported	Not reported	Not reported
Capron 1979 [51]	Case report	63	M	UC-flare	Factor V, factor VIII, antithrombin III	Normal	Not reported	Esophageal varices

## Discussion

Crohn’s disease is linked to a variety of extraintestinal complications. Oral aphthous ulcers, peripheral arthritis, erythema nodosum, and episcleritis are frequently associated with active intestinal disease. Whereas uveitis and ankylosing spondylitis are usually unrelated to disease activity, pyoderma gangrenosum and primary sclerosing cholangitis have a questionable relationship to disease activity [9]. Venous thromboembolic events are fearsome manifestations that are related to disease activity and associated with significant morbidity and mortality [9]. Deep vein thrombosis (DVT) is the most prevalent thrombotic event, followed by pulmonary embolism (PE). The relative risk of thrombotic events in patients with inflammatory bowel disease was 2.03 [10]. Although inflammatory bowel disease treatment options have improved over the last three decades [11], thrombotic events among hospitalized individuals with inflammatory bowel disease continued to rise [12]. The overall thrombotic risk did not differ between sexes or between individuals who have ulcerative colitis or Crohn’s disease [13]. There have been very few reports of portal vein thrombosis in the context of inflammatory bowel disease. The presenting indications, symptoms, and laboratory data are all extremely nonspecific, and a PVT diagnosis is nearly always made by chance. It is important to note that PVT is related to disease activity, particularly IBD flare. We found that portal vein thrombosis affects both men and women, with a small male predominance. It is also more frequent in individuals with ulcerative colitis than in those with Crohn’s disease. It is a rare complication in Crohn’s disease, identified in only 14 cases. Hypercoagulability testing in a subset of patients (around half) revealed inherited or acquired hypercoagulability factors in some, with antiphospholipid antibodies and factor V Leiden mutation being the most common. Treatment for thrombosis in Crohn’s disease involves tailored anticoagulation (heparin, warfarin, DOACs) or even surgery, with outcomes ranging from successful resolution to bleeding or death. However, limitations include the use of case reports and retrospective studies, and the small number of CD cases, which hinder definitive conclusions. There are no recommendations for thrombophilia screening in cases of portal vein thrombosis; many reports, including ours, have included thrombophilia testing. Naymagon *et al*. suggested that thrombophilia testing is not required in cases of clearly triggered PVT, such as after recent surgery or in the setting of a recent or active intraabdominal infection or IBD-flare [1]; moreover, he suggested that thrombophilia testing should be undertaken if PVT is not induced, such as spontaneous PVT in an otherwise stable and inactive IBD patient, or patients with a history of previous venous thromboembolism or unexplained blood count abnormalities [1]. Furthermore, testing for antiphospholipid syndrome and paroxysmal nocturnal hemoglobinuria may affect management and should be considered in certain conditions, such as a history of autoimmune disease or arterial thrombosis for antiphospholipid syndrome and unexplained cytopenia or evidence of intravascular hemolysis for paroxysmal nocturnal hemoglobinuria. Other thrombophilia testing are often unnecessary because the results have little impact on therapy [1]. A mutation of JAK2 could be detected in splanchnic vein thrombosis and thus provide a marker of latent myeloproliferative neoplasms (MPNs), which are a major primary cause of abdominal vein thrombosis [14]. MPNs are made up of three key rare diseases: (1) polycythemia vera, which leads to an elevation in all blood cells, especially red blood cells; (2) essential thrombocythemia, which leads to an increase in platelets; and (3) primary myelofibrosis, a bone marrow disorder that leads to defects in blood cell production [14, 15]. MPNs were diagnosed through a variety of criteria, including the typical alterations in peripheral blood cells [4], as she had chronic active CD with possible previous CD-flare and a normal blood profile which excludes MPNs [1, 14, 15]. We screened for antiphospholipid syndrome antibodies because the patient was a young female with a significant thrombotic event without a clear relationship with a Crohn’s disease flare. Although the lupus anticoagulant (LA1, LA2) was initially positive, it was found to be negative 12 weeks later. The explanations for the false positive in our instance were anticoagulant treatment, including therapy with LWMH, which is indicated to every patient admitted to the hospital with inflammatory bowel disease, and later warfarin for the management of portal vein thrombosis [3, 6, 13]. For PVT management, literature was unclear concerning the selection of anticoagulants. Most patients who require anticoagulation are started on LMWH, or unfractionated heparin, and then switched to vitamin K antagonists (VKAs) to maintain a goal international normalization rate of 2–3. While VKAs can be substituted orally with direct oral anticoagulants (DOACs) or novel oral anticoagulants (NOACs). These medications do not require monitoring of the INR because of their speedier onset of action and lesser risk of bleeding. DOACs are just as effective as VKAs for treating deep vein thrombosis, pulmonary embolism, and stroke prevention in patients with atrial fibrillation, and may be considered owing to potentially less frequent monitoring needs and a fixed dosing regimen, which could enhance medication adherence. However, owing to unbalanced hemostasis, patients with cirrhosis have been excluded from most trials. Our case was portal hypertension without cirrhosis; therefore, DOACs or NOACs are not contraindicated after excluding antiphospholipid syndrome. For Crohn’s disease treatment, ustekinumab was more suitable than tumor necrosis factor inhibitors (anti-TNFα), as ustekinumab had low immunogenicity (generating antidrug antibodies), so it is feasible to avoid a combination of azathioprine and ustekinumab, in contrast to anti-TNF treatment, which necessitates such a combination [3, 6, 7, 9]. Ustekinumab helped to eliminate the drug interactions of azathioprine and warfarin, note that warfarin was the only therapeutic option owing to the initial diagnosis of antiphospholipid syndrome. In addition, ustekinumab had the lowest rate of serious infections among the biological treatments [7]. Esophageal varices primary prophylaxis is not required, as primary prophylaxis must be initiated upon the detection of high-risk varices, such as small varices with red signs, medium or large varices regardless of Child–Pugh classification, or small varices in patients classified as Child–Pugh C [16]. It is possible to discontinue anticoagulant treatment after a year, whether or not portal vein recanalization occurs, because a longer period of anticoagulant treatment is unlikely to enhance the probability of recanalization if it does not occur after a year [4].

## Conclusion

PVT symptoms are similar to the symptoms of an inflammatory bowel disease flare. Initial tests for antiphospholipid syndrome were falsely positive [17]. The wise choice of ustekinumab as the first-line biological treatment, which aided in weaning off azathioprine, led to avoiding azathioprine–warfarin interactions. Using DOACs or NOACs for the management of portal vein thrombosis in case of portal vein hypertension. Finally, the management of esophageal varices in the context of anticoagulant treatment. The use of portal vein Doppler ultrasound, particularly during flare-ups of inflammatory bowel disease, may contribute to the diagnosis and management of this uncommon complication.

## Data Availability

Not applicable.

## Abbreviations

PVT: Portal vein thrombosis
IBD: Inflammatory bowel disease
CD: Crohn’s disease
CRP: C-reactive protein
INR: International normalized ratio
VKAs: K antagonists
CT: Computed tomography
SC: Subcutaneous
APS: Antiphospholipid syndrome
DOAC: Direct oral anticoagulants
NOAC: Novel oral anticoagulants
LMWH: Low molecular weight heparin
HbsAg: Hepatitis B surface antigen
HbsAb: Hepatitis B surface antibody
HbcAb: Hepatitis B core antibody
